# Novel Bi-Modular GH19 Chitinase with Broad pH Stability from a Fibrolytic Intestinal Symbiont of *Eisenia fetida*, *Cellulosimicrobium funkei* HY-13

**DOI:** 10.3390/biom11111735

**Published:** 2021-11-21

**Authors:** Lu Bai, Jonghoon Kim, Kwang-Hee Son, Chung-Wook Chung, Dong-Ha Shin, Bon-Hwan Ku, Do Young Kim, Ho-Yong Park

**Affiliations:** 1 Department of Biotechnology, KRIBB School of Bioscience, Korea University of Science and Technology (UST), Daejeon 34113, Korea; bl127@kribb.re.kr; 2 Industrial Bio-Materials Research Center, KRIBB, Daejeon 34141, Korea; kjh1018@kribb.re.kr (J.K.); sonkh@kribb.re.kr (K.-H.S.); 3 Department of Biological Sciences, Andong National University, Andong 36729, Korea; chung1409@anu.ac.kr; 4 Insect Biotech Co. Ltd., Daejeon 34054, Korea; dhshin@insectbiotech.co.kr (D.-H.S.); balmania@insectbiotech.co.kr (B.-H.K.)

**Keywords:** *Cellulosimicrobium funkei*, intestinal symbiont, *Eisenia fetida*, GH19, endo-type chitinase

## Abstract

Endo-type chitinase is the principal enzyme involved in the breakdown of *N*-acetyl-d-glucosamine-based oligomeric and polymeric materials through hydrolysis. The gene (966-bp) encoding a novel endo-type chitinase (ChiJ), which is comprised of an N-terminal chitin-binding domain type 3 and a C-terminal catalytic glycoside hydrolase family 19 domain, was identified from a fibrolytic intestinal symbiont of the earthworm *Eisenia fetida*, *Cellulosimicrobium funkei* HY-13. The highest endochitinase activity of the recombinant enzyme (rChiJ: 30.0 kDa) toward colloidal shrimp shell chitin was found at pH 5.5 and 55 °C and was considerably stable in a wide pH range (3.5–11.0). The enzyme exhibited the highest biocatalytic activity (338.8 U/mg) toward ethylene glycol chitin, preferentially degrading chitin polymers in the following order: ethylene glycol chitin > colloidal shrimp shell chitin > colloidal crab shell chitin. The enzymatic hydrolysis of *N*-acetyl-β-d-chitooligosaccharides with a degree of polymerization from two to six and colloidal shrimp shell chitin yielded primarily *N,N′*-diacetyl-β-d-chitobiose together with a small amount of *N*-acetyl-d-glucosamine. The high chitin-degrading ability of inverting rChiJ with broad pH stability suggests that it can be exploited as a suitable biocatalyst for the preparation of *N,N′*-diacetyl-β-d-chitobiose, which has been shown to alleviate metabolic dysfunction associated with type 2 diabetes.

## 1. Introduction

Chitin is a renewable biopolymer that is extensively distributed in various ecosystems as the second most abundant structural polysaccharide after β-1,4-d-glucan. This insoluble polysaccharide, which is comprised of *N*-acetyl-d-glucosamine (GlcNAc) repeating units combined by β-1,4-d-glycosidic linkages, is present in the cell walls of fungi and the exoskeletons of insects and crustaceans, including shrimp, krill, and crabs [1,2].

In nature, the biological recycling of chitin polysaccharides is predominantly carried out by a variety of indigenous chitinolytic bacterial and fungal species [3,4]. They generally produce endo-type chitinases (EC 3.2.1.14) and exo-type chitinases (EC 3.2.1.52), such as chitobiosidases and *N*-acetyl-β-d-glucosaminidases, for the cooperative deconstruction of chitins to low molecular weight substances including *N*-acetyl-β-d-chitooligosaccharides (*N*-acetyl-β-d-COSs) and GlcNAc [2,5]. Of the chitinolytic biocatalysts, endo-type chitinases are essential enzymes responsible for the degradation of chitins and, therefore, have attracted much attention as potential candidates for biocontrol of fungal pathogens, the preparation of single cell proteins and *N*-acetyl-β-d-COSs, and anti-fungal therapy, respectively, in agricultural, nutritional, biotechnological, and medical applications [2,4,6]. They are currently classified into four glycoside hydrolase (GH) families (18, 19, 23, and 48) according to their primary sequence similarities (http://www.cazy.org/Glycoside-Hydrolases.html, accessed on 12 May 2021). However, most of the known endo-type chitinases are distributed between GH families 18 and 19. Retaining GH18 chitinases have a (β/α)_8_-barrel structure, while GH19 chitinases with a lysozyme fold are inverting enzymes that hydrolyze β-1,4-d-glycosidic linkages via a single displacement mechanism [7].

Invertebrate herbivores such as earthworms [8,9,10], termites [11,12], mole crickets [13,14], and beetles [15,16] have been reported to contain a variety of carbohydrate polymer-degrading bacteria in their intestines that may participate in either the digestion of plant-based diets, the inhibition of fungal growth, or both. Accordingly, these intestinal microbes generally produce diverse GH enzymes (either cellulases, hemicellulases, chitinases, or both), exhibiting distinct molecular and biocatalytic characteristics for the complete deconstruction of the corresponding polysaccharides [17,18]. Like other invertebrate-symbiotic bacteria [9,11,13,18], *Cellulosimicrobium funkei* HY-13, which is one of the representative lignocellulolytic exo-symbionts isolated from the digestive tract of the earthworm *Eisenia fetida*, has been recently shown to possess at least seven extracellular GH enzymes exhibiting unique degradation activities for plant biomasses [8,10,19,20,21,22,23]. Thus, in this study, to explore a novel endo-type chitinase with distinctive biocatalytic activity from *C. funkei* HY-13 and better understand its carbohydrate-degrading system, we identified the ChiJ gene encoding an acidic, bi-modular GH19 chitinase from its complete genome sequence. Herein, we describe the molecular and functional characteristics of a novel *N*,*N*′-diacetyl-β-d-chitobiose [(GlcNAc)_2_] and GlcNAc-releasing GH19 endochitinase with an N-terminal chitin-binding domain type 3 (ChtBD3) from *C. funkei* HY-13.

## 2. Materials and Methods

### 2.1. Substrate Compounds

Flake chitin from crab shells, flake chitin from shrimp shells, ethylene glycol chitin, Avicel PH-101, carboxymethylcellulose, soluble starch, locust bean gum, beechwood xylan, and lignin were obtained from Sigma-Aldrich (St. Louis, MO, USA). A series of *N*-acetyl-β-d-COSs of (GlcNAc)_2_ to (GlcNAc)_6_, ivory nut mannan, and wheat arabinoxylan was purchased from Megazyme International Ireland Ltd. (Wicklow, Ireland). Flake chitosan from crab shells and GlcNAc were provided by USB Co. (Cleveland, OH, USA) and TCI Co., Ltd. (Tokyo, Japan), respectively.

### 2.2. Cloning of the Chitinase (ChiJ) Gene

For the recombinant production of mature ChiJ proteins in *Escherichia coli* BL21, the encoding gene with an NdeI restriction site in the N-terminus and a HindIII restriction site in the C-terminus was first amplified by polymerase chain reaction (PCR) using *C. funkei* HY-13 genomic DNA as a template and a pair of oligonucleotide primers [ChiJ-F (5’-CATATGGCGACCTGCGCCCCCGC-3’) and ChiJ-R (5’-AAGCTTTCAGCAGGACA- GGTTCGAGCCCGT-3’)]. In this case, the PCR mixture (50 μL) consisted of 2.5 units of FastStart Taq DNA polymerase (Roche, Basel, Switzerland), 2.5 mM of each dNTP, 2 pmol of each primer, 20 ng of template DNA, a GC-rich solution, and a PCR buffer. The PCR reaction was performed using a PCR thermal cycler (TaKaRa, Kyoto, Japan), and the initial template denaturation was accomplished for 4 min at 95 °C, followed by 35 cycles of 30 s at 95 °C, 30 s at 63.5 °C, and 1 min at 72 °C. The amplified products were then separated by agarose gel (1.2%) electrophoresis, after which the desired gene products were purified employing a NucleoSpin Gel and PCR Clean-up (Macherey-Nagel, Düren, Germany). The isolated gene products (834 bp) were then inserted into a pGEM-T easy vector (Promega, Madison, WI, USA) by a ligation reaction. After transformation of the ligation mixture into *E. coli* DH5α, the pGEM-T easy/*chiJ* vectors were isolated from the recombinant cells by employing a NucleoSpin Plasmid (Macherey-Nagel, Düren, Germany) and thereafter digested with the aforementioned endonucleases to generate *chiJ* fragments with the corresponding sticky ends. The resulting *chiJ* fragments were purified and then cloned into a pET-28a(+) vector (Novagen, Darmstadt, Germany) with the same ends, followed by introducing the constructed pET-28a(+)/*chiJ* vectors into *E. coli* BL21.

### 2.3. Production and Purification of Recombinant Chitinase (rChiJ)

To overproduce rChiJ proteins, the recombinant *E. coli* BL21 cells harboring pET- 28a(+)/*chiJ* were cultivated using a 5-L baffled flask with 1 L of Luria-Bertani broth (BD Difco, Franklin Lakes, NJ, USA) and 25 mg/L of kanamycin in a rotary shaker (150 rpm) for 12 h at 30 °C. ChiJ gene overexpression was induced by adding 1 mM isopropyl β-d-1-thiogalactopyranoside (IPTG) after the optical density of the culture reached approximately 0.45 at 600 nm. Following cultivation, the rChiJ-expressing cells were recovered from the culture broth by centrifugation (5000× *g*) for 20 min at 4 °C and then stored at −70 °C for 3 h for further analysis. To purify (His)_6_-tagged rChiJ proteins, the frozen cells were thoroughly resuspended in binding buffer [0.5 M NaCl, 20 mM imidazole, and 20 mM sodium phosphate (pH 7.4)] and disrupted by sonication. Next, the recombinant enzymes accumulated intracellularly as inactive inclusion bodies were harvested by centrifugation (13,000× *g*) for 20 min at 4 °C and subsequently solubilized in binding buffer [0.5 M NaCl, 5 mM imidazole, 1 mM 2-mercaptoethanol, 6 M guanidine hydrochloride, and 20 mM Tris-HCl (pH 8.0)] for affinity purification. The inactive (His)_6_-tagged recombinant enzymes were then isolated in an active form by on-column refolding employing a HisTrap HP column (5 mL) (GE Healthcare, Uppsala, Sweden) connected to a fast-protein liquid chromatography system (Amersham Pharmacia Biotech, Uppsala, Sweden), according to the manufacturer’s instructions. Elution of the (His)_6_-tagged rChiJ from the column was performed by employing a linear gradient of 20–500 mM imidazole at a flow rate of 2 mL/min. The fractions with high endochitinase activity were selectively recovered, combined, and then desalted with a HiPrep 26/10 desalting column (GE Healthcare, Uppsala, Sweden) using 50 mM sodium phosphate buffer (pH 6.0) as the mobile phase. The active fractions exhibiting high endochitinase activity were collected and subjected to further analysis.

### 2.4. Analysis of Proteins

The relative molecular mass of purified rChiJ was analyzed by sodium dodecyl sulfate-polyacrylamide gel electrophoresis (SDS-PAGE) of its denatured polypeptide in a 12.0% gel. After electrophoresis, the proteins separated by SDS-PAGE were visualized by staining the gel with Coomassie Brilliant Blue R-250. A quantitative assay of the protein concentrations was conducted using Bio-Rad Protein Assay Dye Reagent Concentrate (Bio-Rad Laboratories, Inc., Seoul, Korea), as described elsewhere [24].

### 2.5. Enzyme Assays

The chitin-degrading activity of rChiJ was routinely assayed by quantitating the amount of reducing sugars produced after the biocatalytic degradation of colloidal chitin from shrimp shells using the 3,5-dinitrosalicylic acid (DNS) reagent and GlcNAc as a standard. In this study, colloidal chitin was prepared by suspending approximately 4.0 g of chitin from either crab or shrimp shells with stirring in 35.0–37.0% HCl for 50 min, after which 1.0 L of cold distilled water was carefully added. The formed colloids were recovered by centrifugation (5000× *g*) for 20 min at 4 °C, and then the precipitates were washed with distilled water five times. Following sterilization by autoclaving, the prepared colloidal suspension was directly used for endochitinase assays. The standard reaction mixture (0.5 mL) contained 0.5% colloidal shrimp shell chitin and enzyme solution diluted (0.05 mL) in 50 mM sodium acetate buffer (pH 5.5). The biocatalytic reaction of colloidal chitin proceeded at 55 °C for 10 min. One unit (U) of endochitinase activity for a chitin substrate was defined as the amount of rChiJ required to release 1 μmol of reducing sugar per min under standard assay conditions.

### 2.6. Effects of pH, Temperature, and Chemicals on Endochitinase Activity

The effect of pH on the endochitinase activity of rChiJ was estimated by subjecting the recombinant enzymes to a pH range of 3.5 to 11.0 at 55 °C for 10 min, employing the following buffer systems (50 mM): sodium acetate (pH 3.5–5.5), sodium phosphate (pH 5.5–7.5), Tris-HCl (pH 7.5–9.0), and glycine-NaOH (pH 9.0–11.0). The pH stability of rChiJ was determined after preincubating the enzyme in the aforementioned buffers for 1 h at 4 °C, followed by assessing its residual endochitinase activity. In this case, the enzyme reaction was initiated by the addition of 0.5% colloidal shrimp shell chitin to the reaction mixture. The optimum temperature of rChiJ was examined by reacting the enzyme with colloidal shrimp shell chitin (0.5%) at 20, 25, 30, 35, 40, 45, 50, 55, 60, and 65 °C, respectively, in 50 mM sodium acetate buffer (pH 5.5). The thermal stability of rChiJ was evaluated by measuring its residual endochitinase activity after preincubation of the enzyme at the corresponding temperature for 10, 30, and 60 min, respectively, in 50 mM sodium acetate buffer (pH 5.5). The biocatalytic reaction was initiated by the addition of the colloidal substrate to the assay mixture. The stimulatory or inhibitory effects of metal ions (each 1 mM) and chemical reagents (each 5 mM or 0.5%) on the endochitinase activity of rChiJ was assessed after preincubation of the enzyme at 4 °C for 10 min in 50 mM sodium acetate buffer (pH 5.5) containing the substance of interest.

### 2.7. Analysis of the Hydrolysis Products

The biocatalytic hydrolysis of *N*-acetyl-β-d-COSs [(GlcNAc)_2_ to (GlcNAc)_6_, each 1 mg] and colloidal shrimp shell chitin from was performed by reacting the substrates with purified rChiJ (1 μg) in 0.5 mL of 50 mM sodium acetate buffer (pH 5.5) at 50 °C for 6 h, during which it remained fairly stable. After completion of the hydrolysis, the reaction mixture was boiled for 5 min, followed by analyzing the hydrolysis products by thin-layer chromatography (TLC) using GlcNAc and *N*-acetyl-β-d-COSs [(GlcNAc)_2_ to (GlcNAc)_6_ as standards]. The hydrolysis products were separated by TLC with a silica gel 60 F254 plate (20 × 20 cm, Merck, Darmstadt, Germany) as the stationary phase and 1-butanol/acetic acid/water (2:1:1, *v/v*/*v*) as the developing solvent system. Subsequently, to visualize the formed products, the TLC plate was sprayed with ethanol/sulfuric acid (95:5, *v/v*) and then heated at 100 °C for 10 min.

### 2.8. Binding Assay

The substrate-binding ability of rChiJ was investigated using structurally distinct, water-insoluble polymers that included colloidal shrimp shell chitin, shrimp shell chitin flake, chitosan flake, Avicel PH-101, wheat arabinoxylan, ivory nut mannan, and lignin. For this, the respective hydrophobic polymer in a 1.5 mL Eppendorf tube was first washed four times with sterile distilled water to eliminate any water-soluble compounds and subsequently re-washed with 50 mM sodium acetate buffer (pH 5.5) once more. The appropriately diluted enzyme preparation (5.0 U/mL) was then combined with an equal volume of the prepared polymer, followed by incubating the mixture on ice for 2 h with vigorous stirring every 5 min. After finishing the binding experiments, the supernatants containing rChiJ unbound to the examined polymers were collected by centrifugation (13,000× *g*) for 10 min at 4 °C and subjected directly to the remaining endochitinase activity and protein concentration assays.

## 3. Results and Discussion

### 3.1. Genetic Characterization of the GH19 Chitinase Gene

**Figure 1:**
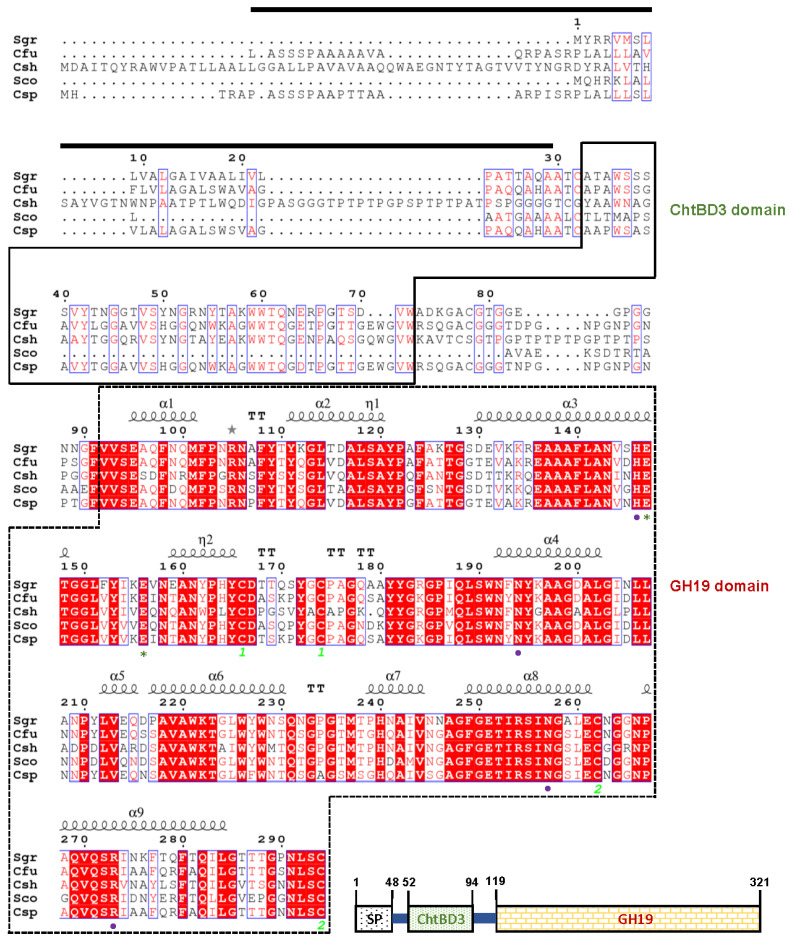
Domain architecture of *Cellulosimicrobium funkei* HY-13 GH19 chitinase (ChiJ) and structure-based sequence alignment of the enzyme and its structural homologs, which was generated using ESPript 3.0 server (https://espript.ibcp.fr/ESPript/ESPript, accessed on 12 May 2021). The first line shows the secondary structure elements (α-helix, squiggle; 3_10_-helix, η; β-turn, TT) of *Streptomyces griseus* HUT6037 GH19 chitinase (PDB code: 1WVU) employed as a template. Sequences (GenBank accession numbers): Sgr: *Streptomyces* *griseus* HUT6037 GH19 chitinase (BAA23739); Cfu: *C. funkei* HY-13 GH19 chitinase (ChiJ) (OK482705); Csh: *Chitiniphilus shinanonensis* GH19 chitinase (BAK53965); Sco: *Streptomyces coelicolor* A3(2) GH19 chitinase (CAD55444); and Csp: *Cellulosimicrobium* sp. TH-20 GH19 chitinase (WP_144679631). The predicted signal peptide is indicated by a black bar. GH19 and ChtBD3 domains are outlined by dotted and solid lines, respectively. Highly conserved amino acid residues that play a key role in biocatalysis are displayed by asterisks. The putative amino acid residues contributing to substrate binding are indicated by four purple closed circles. The disulfide-forming cysteine residues are shown by numbers pairwise.

**Figure 2:**
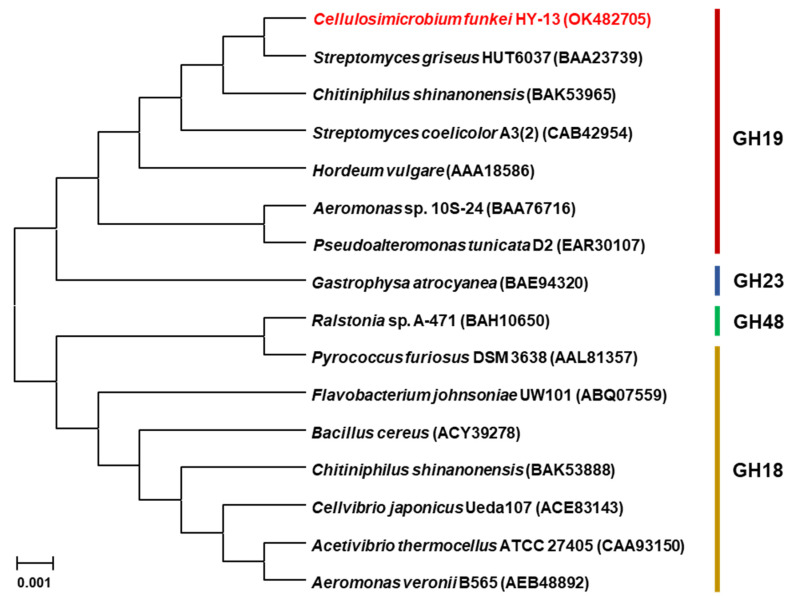
Phylogenetic analysis of *C. funkei* HY-13 GH19 chitinase (ChiJ) and its closely related functional homologs. Multiple alignment of the amino acid sequences was achieved using ClustalW in the MegAlign software (http://www.megasoftware.net, accessed on 12 May 2021). The protein sequence data used for phylogenetic analysis were retrieved from the GenBank database.

The 966-bp ChiJ gene (GenBank accession number: OK482705) coding for an extracellular GH19 chitinase was identified from the whole genome sequence of *C. funkei* HY-13. The nucleotide sequence of *chiJ* was predicted to express a premature protein of 321 amino acids with a calculated pI of 6.56 and a deduced molecular mass of 33,275 Da. However, when analyzed by the SignalP 5.0 server (http://www.cbs.dtu.dk/services/SignalP, accessed on 12 May 2021), the mature ChiJ generated by the post-translational proteolytic cleavage of a signal peptide (from Leu1 to Ala48 in Figure 1) of the premature ChiJ was predicted to be a protein with a calculated pI of 5.78 and a deduced molecular mass of 28,660 Da. Pfam, SMART, and protein BLAST analyses of the primary structure of premature ChiJ displayed that it might be a bi-modular chitinase consisting of a chitin-binding domain type 3 (ChtBD3: from Ala52 to Trp94) in the N-terminus region and a putative catalytic GH19 domain (from Val119 to Cys321) in the C-terminus region (Figure 1). The result of phylogenetic analysis also showed that the primary sequence of ChiJ shared a close evolutionary relationship with that of inverting GH19 chitinases (EC 3.2.1.14) in the phylogenetic tree (Figure 2).

A protein BLAST survey indicated that with a sequence identity of 92.6%, the primary sequence of premature ChiJ was most similar to that of an uncharacterized *Cellulosi-microbium cellulans* GH19 chitinase (GenBank accession number: WP_087470179), which was just identified through a genome survey. Moreover, multiple alignment of the protein sequences revealed that the catalytic GH19 domain of ChiJ shared relatively high sequence identities of 85.5% to 93.6% with that of chitinases from most *Cellulosimicrobium* species available in the National Center for Biotechnology Information (NCBI) database, which has not yet been biocatalytically characterized. In contrast, as exhibited in Figure 1, the GH19 domain of ChiJ was 83.7%, 77.8%, and 69.3% identical to that of *Streptomyces griseus* HUT6037 chitinase (BAA23739) [25], *Streptomyces coelicolor* A3(2) chitinase (CAD55444) [26], and *Chitiniphilus shinanonensis* SAY3^T^ chitinase (BAK53965) [27], respectively, which have been functionally identified. It should also be noted that sequence identities between the ChtBD3 domain of ChiJ and that of *S. griseus* HUT6037 GH19 chitinase (BAA23739) [25] and *C. shinanonensis* SAY3^T^ GH19 chitinase (BAK53965) [27] were assessed to be 53.6% and 53.5%, respectively. In ChiJ, the two putative catalytic residues (Glu174 acting as the proton donor and Glu183 acting as the nucleophile/base) and four conserved substrate-binding site residues (His173, Asn221, Asn284, and Arg300) were predicted in the active site of the enzyme, as exist in other GH19 functional homologs [27,28,29].

The secondary structural features of ChiJ from *C. funkei* HY-13, which were identified using a GH19 chitinase (PDB code: 1WVU) from *S. griseus* HUT6037 as a template, are shown in Figure 1. The structure-based sequence alignment performed using the ESPript 3.0 program showed that the catalytic GH19 domain in ChiJ from *C. funkei* HY-13 was comprised of 9 α-helices, 2 3_10_-helices, and 5 β-turns. In this enzyme, the formation of intramolecular disulfide bonds, which is required for protein folding and stability, was predicted to occur between Cys193 and Cys201 and between Cys289 and Cys321.

### 3.2. Purification and SDS-PAGE Analysis of rChiJ

**Figure 3:**
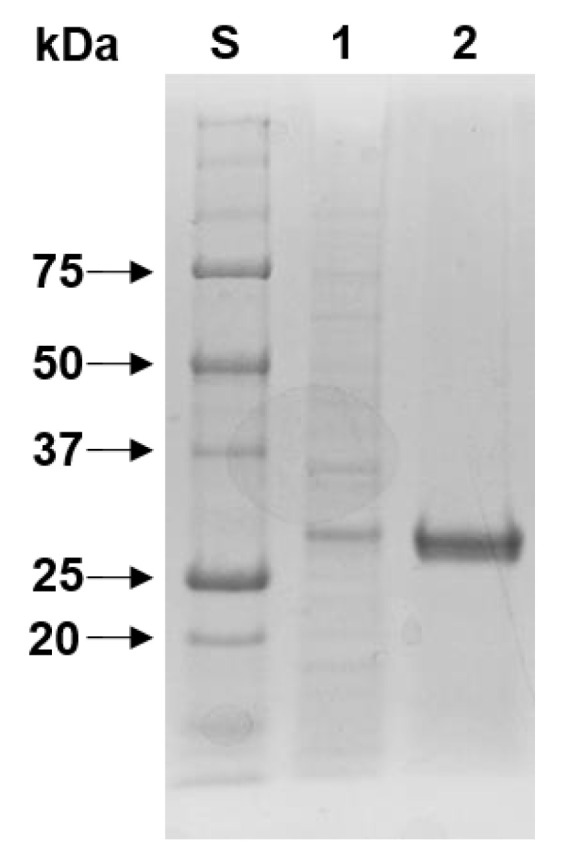
SDS-PAGE of the purified rChiJ after affinity chromatography on HisTrap HP. Lane S, standard marker proteins; lane 1, the total cell lysate of rChiJ-expressing *E. coli* BL21 after IPTG induction; lane 2, purified rChiJ.

Similar to *Vibrio proteolyticus* GH19 chitinase [30] and *Chitinibacter tainanensis* CT01 GH19 chitinase [31], most of the rChiJ proteins overproduced in recombinant *E. coli* BL21 were obtained as inactive inclusion bodies. Therefore, active rChiJ proteins with endochitinase activity were simply purified to electrophoretic homogeneity by an on-column refolding procedure of proteins employing a Ni-NTA column. When determined by SDS-PAGE (Figure 3), the relative molecular mass of purified (His)_6_-tagged rChiJ was estimated to be approximately 30.0 kDa, which was consistent with its deduced molecular mass (30,955 Da). The molecular size of rChiJ was relatively similar to that of *S. griseus* HUT6037 GH19 chitinase (28.5 kDa) [25], *Streptomyces alfalfae* ACCC 40021 GH19 chitinase (29.0 kDa) [29], *Streptomyces cyaneus* SP-27 GH19 chitinase (29.0 kDa) [32], and *Streptomyces sampsonii* XY2-7 GH19 chitinase (30.0 kDa) [33], as listed in Table 1. However, bi-modular rChiJ was assessed to have a much smaller molecular mass than other characterized functional homologs such as *Aeromonas* sp. No. 10S-24 GH19 chitinase (70.0 kDa) [34], *C. shinanonensis* SAY3^T^ GH19 chitinase (41.4 kDa) [27], and *Pseudoalteromonas tunicata* GH19 chitinase (53.5 kDa) [28].

### 3.3. Enzymatic Properties of rChiJ

**Figure 4:**
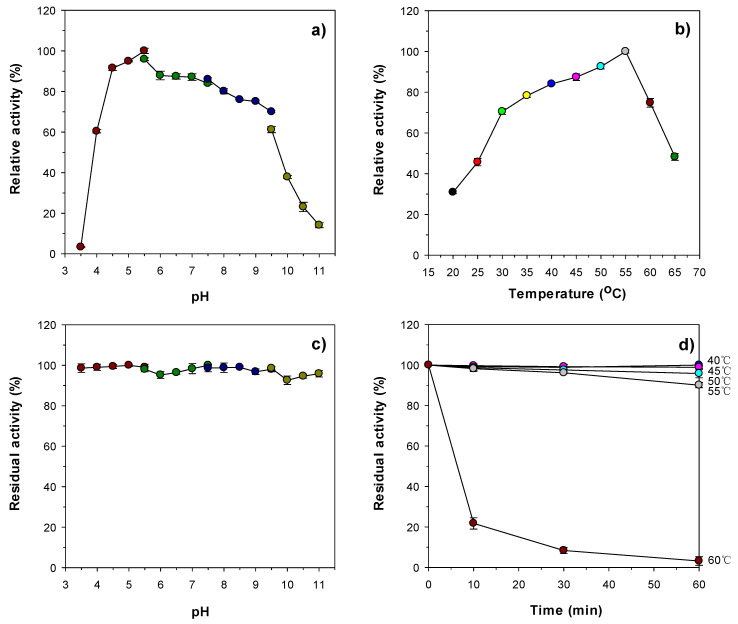
Effects of pH (**a**) and temperature (**b**) on the endochitinase activity of rChiJ and effects of pH (**c**) and temperature (**d**) on the stability of rChiJ. The optimal pH of rChiJ was determined using the following buffers (50 mM): sodium acetate (pH 3.5–5.5), sodium phosphate (pH 5.5–7.5), Tris-HCl (pH 7.5–9.5), and glycine-NaOH (pH 9.5–11.0). The optimal temperature of rChiJ was evaluated at different temperatures (20–65 °C) in 50 mM sodium acetate buffer (pH 5.5). The pH stability of rChiJ was examined at pH 3.5–11.0 after preincubation of the enzyme using the aforementioned buffer systems at 4 °C for 60 min. The thermal stability of rChiJ was assessed by measuring its residual endochitinase activity after preincubation of the enzyme at 40, 45, 50, 55, and 60 °C in 50 mM sodium acetate buffer (pH 5.5) for 10, 30, and 60 min, respectively. The values are mean ± SD of triplicate tests.

When reacted with colloidal chitin from shrimp shells, rChiJ displayed a maximum biocatalytic activity toward the substrate at pH 5.5 and 55 °C (Figure 4a,b). Conversely, its biocatalytic capacity was significantly reduced at pH values lower than 4.5 or higher than 9.0, and at temperatures exceeding 55 °C. It is noteworthy that the enzyme retained over 93% of its original chitin-degrading activity even when preincubated for 1 h in a wide pH range from 3.5 to 11.0 (Figure 4c). It appeared that rChiJ was relatively stable at temperatures below 55 °C for 1 h, but its thermostability was considerably downregulated when preincubated at 60 °C for the same preincubation time (Figure 4d). The optimum pH (5.5) of rChiJ was very similar to that (5.6) of *C. shinanonensis* SAY3^T^ GH19 chitinase, which exhibited the highest degradation activity for colloidal chitin at 50 °C [27]. However, unlike rChiJ, some bacterial GH19 chitinases listed in Table 1 have been reported to be most active toward colloidal chitin at neutral or alkaline pH values [28,29,32,35]. In addition, the optimum temperature (55 °C) of rChiJ was significantly comparable to that of *Aeromonas* sp. No. 10S-24 GH19 chitinase [34], *Streptomyces griseus* MG3 GH19 chitinase [36], *S. alfalfae* ACCC 40021 GH19 chitinase [29], *Nosema bombycis* (Nb) GH19 chitinase [35], and *P. tunicata* GH19 chitinase [28] that most efficiently decomposed colloidal chitin at temperatures below 45 °C. However, similar to rChiJ, *S. griseus* HUT6037 GH19 chitinase [25] and *S. sampsonii* XY2-7 GH19 chitinase [33] were shown to maximally degrade the substrate at 55 °C.

**Figure 5:**
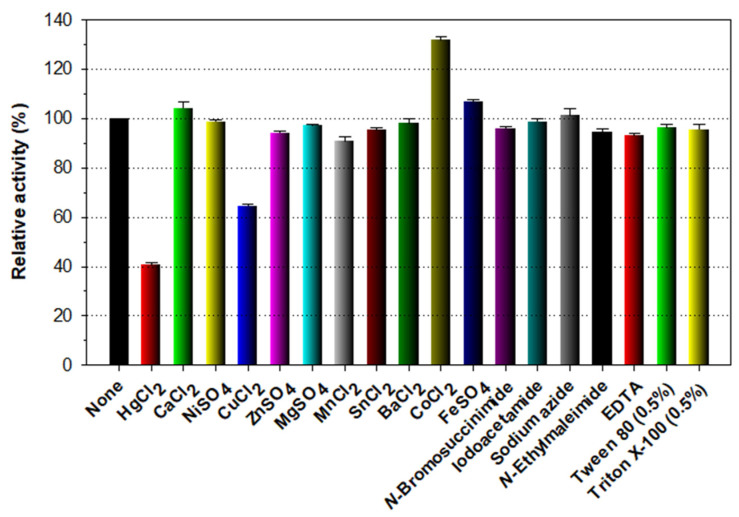
Effects of metal ions (1 mM) and chemical reagents (5 mM) on the endochitinase activity of rChiJ. The values are mean ± SD of triplicate tests.

As displayed in Figure 5, the chitin-degrading activity of rChiJ was hardly affected by the tryptophan (Trp) residue-directed modifier *N*-bromosuccinimide (5 mM) when preincubated in the presence of the chemical reagent. Moreover, the reduction of the original chitin-degrading activity of rChiJ induced by 1 mM Hg^2+^ was measured to be approximately 60%. Taken together, these findings implied that rChiJ was a structurally different enzyme that was less sensitive to Trp-specific chemical compounds compared to other characterized GH19 chitinases [28,29], which were almost completely or significantly inactivated by the chemical modifiers. Hg^2+^ ions and *N*-bromosuccinimide were previously reported to oxidize the indole ring of strictly conserved Trp residues in endo-type glycoside hydrolases, which necessarily take part in enzyme-substrate interaction [37,38]. In the primary structure of ChiJ (Figure 1), the three conserved Trp residues (W78, W79, and W94) in the ChtBD3 domain and four conserved Trp residues (W218, W248, W253, and W255) in the catalytic GH19 domain were predicted to be susceptible to Hg^2+^. Interestingly, the biocatalytic activity of rChiJ for colloidal shrimp shell chitin could be upregulated by 1.3-fold in the presence of 1 mM Co^2+^, whereas its enzyme activity was downregulated by approximately 35% in the presence of 1 mM Cu^2+^. A similar observation was also made with a GH19 chitinase from *S. alfalfae* reacted with colloidal chitin in the presence of 1 mM Cu^2+^ that resulted in an approximate 29% reduction in its original biocatalytic activity, although its biocatalytic activity was greatly stimulated by 1.8-fold in the presence of 1 mM Co^2+^ [29]. However, *P. tunicata* CCUG 44952T GH19 chitinase [28] has been shown to be completely inactivated by 1 mM Cu^2+^, which had almost no influence on the biocatalytic activity of *S. sampsonii* GH19 chitinase [33]. In this study, the inhibitory or stimulatory effect of rChiJ by the tested divalent cations (each 1 mM) including Ca^2+^, Ni^2+^, Zn^2+^, Mg^2+^, Mn^2+^, Sn^2+^, Ba^2+^, and Fe^2+^ was evaluated to be insignificant, as previously shown for *S. sampsonii* GH19 chitinase [33]. Conversely, *Chitinolyticbacter meiyuanensis* SYBC-H1 GH19 exo-type chitinase was proven to be strongly inactivated by 10 mM Zn^2+^ or Fe^2+^ [39]. Moreover, the biocatalytic activity of *P. tunicata* CCUG 44952T GH19 chitinase was considerably downregulated by > 88% in the presence of 1 mM Ni^2+^ or Fe^2+^ [28]. Unlike *S. sampsonii* GH19 chitinase, whose chitin-degrading activity was greatly increased by approximately 1.5-fold by Triton X-100 [33], rChiJ activity was not substantially affected by a non-ionic detergent (Figure 5). Similarly, no considerable inhibition or stimulation of rChiJ was observed by preincubation with EDTA and sulfhydryl reagents, including *N*-ethylmaleimide, sodium azide, and iodoacetamide.

### 3.4. Substrate Specificity

In this study, the substrate specificity of rChiJ was examined employing different types of GlcNAc, d-glucose, d-mannose, and d-xylose-based polysaccharides together with *N*-acetyl-β-d-COSs [(GlcNAc)_2_ to (GlcNAc)_6_]. Of the tested polymeric materials, the enzyme was able to readily depolymerize diverse chitin polysaccharides in the order of ethylene glycol chitin > colloidal chitin from shrimp shells > colloidal chitin from crab shells (Table 2). In contrast, rChiJ did not show any detectable hydrolysis activity toward soluble starch, carboxymethylcellulose, locust bean gum, or beechwood xylan, suggesting that it was a true GH19 endo-type chitinase lacking other additional glycoside hydrolase activities. The specific activity of rChiJ for ethylene glycol chitin, colloidal shrimp shell chitin, and colloidal crab shell chitin was determined to be 338.8 U/mg, 16.0 U/mg, and 8.1 U/mg, respectively. Based on these results, rChiJ was assessed to be most active on ethylene glycol chitin compared to other characterized GH19 chitinases, as described below. Specifically, the biocatalytic activity of rChiJ (338.8 U/mg) toward ethylene glycol chitin was evaluated to be approximately 1.2-fold, 5.8-fold, 13.5-fold, and 376.4-fold higher than that of *S. alfalfae* ACCC 40021 GH19 chitinase (286.6 U/mg) [29], *Nosema bombycis* (Nb) GH19 chitinase (58.6 U/mg) [35], *S. cyaneus* SP-27 GH19 chitinase (25.0 U/mg) [32], and *Aeromonas* sp. No. 10S-24 GH19 chitinase (0.9 U/mg) [34], respectively, toward the same substrate. It should also be noted that the biocatalytic activity of rChiJ (16.0 U/mg) for colloidal chitin was notably superior to that of *P. tunicata* GH19 chitinase (<0.1 U/mg) [28], *Nosema bombycis* (Nb) GH19 chitinase (0.7 U/mg) [35], *S. griseus* MG3 GH19 chitinase (1.8 U/mg) [36], and *C. shinanonensis* SAY3^T^ GH19 chitinase (8.6 U/mg) [27]. On the other hand, the colloidal chitin-degrading activity of rChiJ (16.0 U/mg) was estimated to be approximately 1.8-fold and 1.5-fold lower than that of *S. alfalfae* ACCC 40021 GH19 chitinase (28.4 U/mg) [29] and *S. griseus* HUT6037 GH19 chitinase (24.5 U/mg) [25], respectively. Taken together, the results suggest that rChiJ is a novel endo-type chitinase with distinct substrate specificity compared to other known GH19 functional homologs (EC 3.2.1.14) (Table 1).

**Figure 6:**
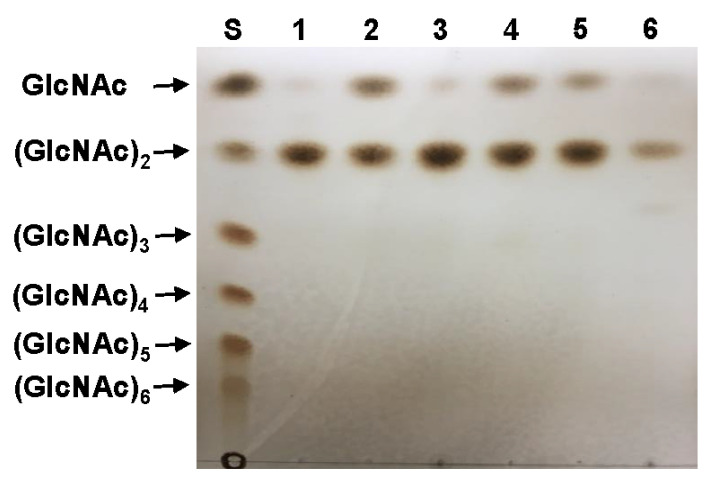
TLC analysis of the hydrolysis products of *N*-acetyl-β-d-COSs of (GlcNAc)_2_ to (GlcNAc)_6_ and colloidal shrimp shell chitin by rChiJ: Lane S, standard markers [GlcNAc to (GlcNAc)_6_]; lanes of 1 to 5, the reaction of rChiJ with *N*-acetyl-β-d-COSs [(GlcNAc)_2_ to (GlcNAc)_6_, each 1 mg]; lane 6, the reaction of rChiJ with colloidal shrimp shell chitin.

It has been previously demonstrated that *S. alfalfae* ACCC 40021 GH19 chitinase [29], *S. sampsonii* GH19 chitinase [33], and *Nosema bombycis* (Nb) GH19 chitinase [35] can pre-ferentially catalyze the hydrolysis of colloidal chitin, (GlcNAc)_3_, (GlcNAc)_5_, and (GlcNAc)_6_ to GlcNAc together with (GlcNAc)_2_ as the major end product, in a time-dependent manner. Moreover, the above enzymes were reported to exclusively hydrolyze (GlcNAc)_4_ to (GlcNAc)_2_, whereas they did not show any cleavage activity toward (GlcNAc)_2_. Nevertheless, compared to the aforementioned enzymes [29,33,35], the results of TLC analysis clearly revealed that rChiJ could hydrolyze *N*-acetyl-β-d-COSs [(GlcNAc)_2_ to (GlcNAc)_6_] and colloidal shrimp shell chitin to (GlcNAc)_2_ molecules as the major end product and GlcNAc as the minor end product (Figure 6). The degradation patterns of (GlcNAc)_6_ and colloidal chitin by rChiJ were significantly comparable to those of the same chitinous substrates by some bacterial GH19 endo-type chitinases [30,31,34]. Specifically, *Aeromonas* sp. No. 10S-24 GH19 chitinase [34] and *V. proteolyticus* GH19 chitinase [30] catalyzed the degradation of (GlcNAc)_6_ and colloidal chitin, respectively, to (GlcNAc)_4_, (GlcNAc)_3_, and (GlcNAc)_2_. It has also been reported that the deconstruction of colloidal chitin by *C. tainanensis* CT01 GH19 chitinase yields (GlcNAc)_2_ as the major end product together with GlcNAc and (GlcNAc)_3_ as the minor end products [31]. In Figure 6, the susceptibility of (GlcNAc)_2_ to the enzyme was likely to be very weak because only a small amount of GlcNAc as the hydrolysis product was observed to be released from the substrate. Moreover, the amount of GlcNAc formed by the enzymatic hydrolysis of (GlcNAc)_4_ and colloidal shrimp shell chitin was significantly lower than that of GlcNAc released from (GlcNAc)_3_, (GlcNAc)_5_, and (GlcNAc)_6_ in the biocatalytic reactions. Thus, it is believed that (GlcNAc)_4_ and colloidal shrimp shell chitin were predominantly cleaved in the middle and at the internal β-1,4-d-glycosidic linkages, respectively, within the backbone and related to the release of even-numbered *N*-acetyl-β-d-COSs. Also, it is assumed that at the initial reaction stage, the cleavage of (GlcNAc)_5_ and (GlcNAc)_6_ by rChiJ might have occurred at the second and third β-1,4-d-glycosidic linkages from the non-reducing end to yield (GlcNAc)_2_ together with (GlcNAc)_3_, which was then further hydrolyzed to GlcNAc and (GlcNAc)_2_. The formation of longer *N*-acetyl-β-d-COSs from the substrate molecules used in the biocatalytic reactions was not observed, indicating that rChiJ lacked transglycosylation activity (Figure 6). Taken together, these findings strongly suggested that rChiJ might be a novel GH19 endo-type chitinase without transglycosylation activity, which was distinguished from other GH19 functional homologs [29,30,31,33,34,35] in the hydrolysis patterns of GlcNAc-based materials.

### 3.5. Binding Capacity of rChiJ to Hydrophobic Materials

**Figure 7:**
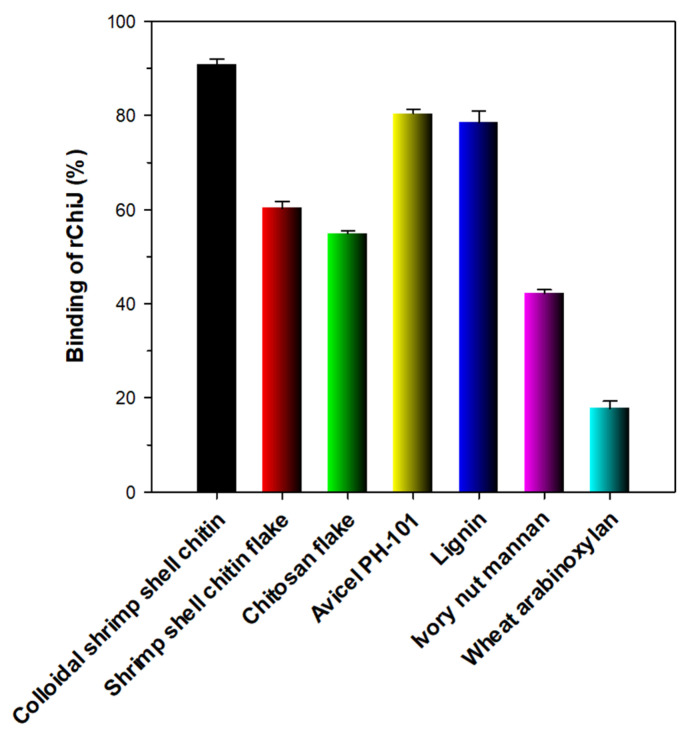
Binding of rChiJ to insoluble polymers. The values are mean ± SD of triplicate tests.

For the efficient deconstruction of GlcNAc-based polymers, endo-type chitinases should be tightly attached to the surface of the target polymeric materials in the initial stage of enzyme-substrate interaction. Thus, we examined the binding capacity of rChiJ to various hydrophobic materials, including cellulosic and hemicellulosic polysaccharides, lignin, chitosan, and chitin from shrimp shells (Figure 7). Of the evaluated insoluble materials, rChiJ with an N-terminal ChtBD3 domain as a substrate-binding motif displayed the highest binding affinity (>90%) to colloidal shrimp shell chitin, while its binding capacity to shrimp shell chitin flake was assessed to be approximately 60% under the same reaction conditions. It is considered that this phenomenon might be due to the much smaller particle size of colloidal shrimp shell chitin compared to shrimp shell chitin flake, which may cause a great increase in the surface area of the same polysaccharide. The binding ability (60%) of rChiJ to shrimp shell chitin flake was very similar to that (61%) of a GH19 chitinase with two N-terminal chitin-binding domains from *C. shinanonensis* [27] to chitin flake. In particular, the deletion of substrate-binding domains in *C. shinanonensis* GH19 chitinase was shown to give rise to a significant reduction (88%) in its chitin flake-binding capacity, indicative of the functional role of chitin-binding domains in enzyme-substrate interaction [27]. In this study, it is also worth noting that rChiJ showed relatively strong binding ability (>80%) to Avicel PH-101 and lignin with structurally different microstructures. However, the binding affinity of rChiJ to ivory nut mannan and wheat arabinoxylan was observed to be relatively lower than 45%.

## 4. Conclusions

The bi-modular GH19 chitinase (ChiJ) with an N-terminal ChtBD3 domain from *C. funkei* HY-13 is a novel endo-type biocatalyst showing distinct characteristics in its amino acid sequence, pH and thermal properties, sensitivity to divalent cations, substrate specificity, and hydrolysis of chitinous materials. Considering its high endochitinase activity toward different chitin polysaccharides, this enzyme with broad pH stability can be exploited as a suitable candidate for the preparation of (GlcNAc)_2_, which was recently shown to ameliorate metabolic dysfunction associated with type 2 diabetes [40]. Similar to some anti-fungal GH19 chitinases that effectively inhibited the growth of fungal pathogens [28,29,32], rChiJ showing high chitin-degrading capacity is also expected to be a suitable anti-fungal agent for applications in the food and pharmaceutical industries through synergistic combination with a highly active, anti-fungal GH64 endo-β-1,3- glucanase from *C. funkei* HY-13 [10].

## Figures and Tables

**Table 1:** Enzymatic characteristics of GH19 chitinases (E.C.3.2.1.14) active for chitin polysaccharides.

Strain	Enzyme	M*_r_* (kDa)	Optimum pH	Optimum Temp. (°C)	Specific Activity (U/mg)	Reference
*Cellulosimicrobium funkei* HY-13	rChiJ	30.0	5.5	55	338.8 ^a^, 16.0 ^b^	This study
*Streptomyces griseus* HUT6037	chitinase C	28.5	4.5–6.0	55	255.0 ^a^, 24.5 ^b^	[25]
*Aeromonas* sp. No. 10S-24	chitinase	70.0	5.0	40	0.9 ^a^	[34]
*Nosema bombycis* (Nb)	NbchiA	NI	7.0	40	58.6 ^a^, 0.7 ^b^	[35]
*Streptomyces griseus* MG3	ChiIS	29.0	5.0–7.0	45	1.8 ^b^	[36]
*Streptomyces coelicolor* A3(2)	Chi19F	<30.0	6.0–7.0	50	NI ^c^	[26]
*Chitiniphilus shinanonensis* SAY3^T^	rChiN	41.4	5.6	50	8.6 ^b^	[27]
*Pseudoalteromonas tunicata*	*Pt*Chi19p	53.5	7.5	43	<0.1 ^b^	[28]
*Streptomyces cyaneus* SP-27	chitinase A	29.0	7.0	60	25.0 ^a^	[32]
*Streptomyces sampsonii* XY2-7	SsChi28	30.0	6.0	55	222.3 ^a^, 20.1 ^b^	[33]
*Streptomyces alfalfae* ACCC 40021	*Sa*ChiB	29.0	8.0	45	286.6 ^a^, 28.4 ^b^	[29]

One unit (U) of endochitinase activity is defined as the amount of protein required to produce 1 μmol of reducing sugar per min. ^a^ Specific enzyme activity toward ethylene glycol chitin; ^b^ Specific enzyme activity toward colloidal chitin; ^c^ Not indicated.

**Table 2:** Biocatalytic activity of rChiJ for different polysaccharides.

Substrate	Specific activity (U/mg) ^a^ of rChiJ	Relative activity (%) of rChiJ
Ethylene glycol chitin	338.8 ± 1.2	100.0
Colloidal shrimp shell chitin	16.0 ± 0.3	4.7
Colloidal crab shell chitin	8.1 ± 0.2	2.4
Carboxymethylcellulose	ND ^b^	-
Soluble starch	ND	-
Beechwood xylan	ND	-
Locust bean gum	ND	-

The values are mean ± SD of triplicate tests; ^a^ Specific activity was obtained from the three repeated experiments; ^b^ Not detected.

## Data Availability

Not applicable.
